# Expression and Function of Kisspeptin during Mouse Decidualization

**DOI:** 10.1371/journal.pone.0097647

**Published:** 2014-05-15

**Authors:** Peng Zhang, Min Tang, Ting Zhong, Yan Lin, Teng Zong, Chengxue Zhong, BaoPing Zhang, Min Ren, HaiBin Kuang

**Affiliations:** 1 Department of Physiology, School of Medicine, Nanchang University, Nanchang, Jiangxi, China; 2 Department of Cell Biology, School of Medicine, Nanchang University, Nanchang, Jiangxi, China; 3 Department of Obstetrics and Gynecology, Hospital of Jixi Province People, Nanchang, Jiangxi, China; 4 Department of Reproductive Medicine, First Affiliated Hospital of Gannan Medical University, Ganzhou, Jiangxi, China; State Key Laboratory of Reproductive Biology, Institute of Zoology, Chinese Academy of Sciences, China

## Abstract

**Background:**

Plasma kisspeptin levels dramatically increased during the first trimester of human pregnancy, which is similar to pregnancy specific glycoprotein-human chorionic gonadotropin. However, its particular role in the implantation and decidualization has not been fully unraveled. Here, the study was conducted to investigate the expression and function of kisspeptin in mouse uterus during early pregnancy and decidualization.

**Methodology/Principal Findings:**

Quantitative PCR results demonstrated that Kiss1 and GPR54 mRNA levels showed dynamic increase in the mouse uterus during early pregnancy and artificially induced decidualization in vivo. KISS-1 and GPR54 proteins were spatiotemporally expressed in decidualizing stromal cells in intact pregnant females, as well as in pseudopregnant mice undergoing artificially induced decidualization. In the ovariectomized mouse uterus, the expression of Kiss1 mRNA was upregulated after progesterone or/and estradiol treatment. Moreover, in a stromal cell culture model, the expression of Kiss1 and GPR54 mRNA gradually rise with the progression of stromal cell decidualization, whereas the attenuated expression of Kiss1 using small interfering RNA approaches significantly blocked the progression of stromal cell decidualization.

**Conclusion:**

our results demonstrated that Kiss1/GPR54 system was involved in promoting uterine decidualization during early pregnancy in mice.

## Introduction

The establishment of successful embryo implantation requires reciprocal interactions between the receptive uterus and activated state of the blastocyst [1]. The onset of blastocyst attachment with the uterine luminal epithelium occurs at 2200–2300 h on day 4 of pregnancy in the mouse [1], [2]. It is followed by the proliferation and the differentiation of stromal cells surrounding implanting blastocyst into decidual cells with polyploidy [3], [4]. In rodent, this differentiation process, termed decidualization, is critical for the establishment of normal pregnancy and fetal development [2]. However, the molecular mechanisms that are involved in these events remain poorly understood. Recent studies have suggested that kisspeptins may play a role in the implantation and pregnancy [5], [6] in addition to their recognized roles in the central control of the gonadotrophic axis [6], [7], [8] and tumor metastasis [9], [10], [11].

Kisspeptin, a neuropeptide hormone encoded by Kiss1 gene, was originally discovered in 1996 from melanoma cell lines with different metastatic capacity [9]. The Kiss1 gene encoded a number of structurally-related peptides (such as kisspeptin-54, 14, 13 and 10), which were derived from the differential proteolytic processing of a 145 amino acid precursor and globally termed as kisspeptins [6], [12]. Kisspeptins are endogenous ligands for the orphan G protein-coupled receptor named GPR54 in rat [10] and AXOR12 or hOT7T175 in humans [11], and all kisspeptins are able to bind GPR54, with kisspeptin-10 having maximal activity at the receptor level [13]. In 2003, two research groups independently reported that deletions and inactivating mutations of GPR54 gene are linked to absence of puberty onset and hypogonadotrophic hypogonadism in humans[8], [14], which was reproduced in GPR54 knock-out mice[14], [15]. These observations revealed that Kiss1/GPR54 system was an essential gatekeeper of puberty onset and reproductive function.

During human pregnancy, plasma kisspeptin levels are dramatically elevated and it rose to a peak of 9590±1640 fmol/ml in third trimester. On day 5 after delivery, it returned to nearly nonpregnant state (7.63±1.33 fmol/ml) [16], which is similar to pregnancy specific glycoprotein-human chorionic gonadotropin (hCG). Kisspeptin/GPR54 was shown to express at the feto-maternal interface, and abundant in the syncytiotrophoblast of both normal human placenta [16], [17] and in hydatidiform molar pregnancies [18]. Furthermore, the levels of serum kisspeptin in early pregnancy are associated with intra-uterine growth restriction, recurrent pregnancy loss and preeclampsia [19], [20].In addition, quantitative polymerase chain reactions (PCR) and in situ hybridization analysis indicated that both Kiss1 and GPR54 mRNA were expressed in rat uterus and placenta [21]. GPR54 knockout mice, males and females were incapable of reproducing when paired with fertile littermates [14], [15]. Most recently, our data indicated that kisspeptin could directly augment progesterone secretion in rat luteal cells [22]. However, the physiological relevance of Kiss1/GPR54 during implantation and decidualization remains to be further explored.

In the present study, we investigated that the expression and regulation of Kiss1/GPR54 in mouse uterus during early pregnancy, artificial decidualization and hormonal treatments by quantitative PCR and immunohistochemistry. We also observed whether Kiss1/GPR54 regulated the progression of stromal cell decidualization by short interfering RNA method in vitro.

## Materials and Methods

### Animals and treatments

Adult CD-1 mice were caged in a constant photoperiod (14 h light: 10 h dark cycle). The experimental protocols were approved by the Institutional Animal Care and Use Committee of Nanchang University (Permit Number: NJ20100213). Virgin female mice (weight 25–30 g, 6–8 weeks old) were mated with fertile or vasectomized males of the same strain to induce pregnancy or pseudopregnancy (day 1 =  day of vaginal plug), respectively. Mice were killed to collect uteri at 0900 hours on various days of pregnancy. Pregnancy on days 1–4 were confirmed by recovering embryos from the reproductive tracts. Implantation sites on days 5 and 6 were identified by intravenous injection of 0.1 ml 1% Chicago blue and implantation sites were demarcated by discrete blue bands. The region between two implantation sites was termed as the interimplantation sites.

Artificial decidualization (artificially induced deciduoma) was induced by intraluminal injections of sesame oil (25 µl/mouse) in one uterine horn on day 4 of pseudopregnancy, while the contralateral uninjected horn served as a control. The mice were killed to collect uteri on days 5–8 of pseudopregnancy. Decidualization was confirmed by uterine weight and histological examination of uterine sections.

To determine the effects of steroid hormones on the expression of Kiss1 and GPR54 in uteri, hormonal treatments were initiated 2 weeks after mature female mice were ovariectomized. Progesterone (P4, Sigma) and estradiol-17β (E2, Sigma) were dissolved in sesame oil. Ovariectomized mice were injected with sesame oil (0.1 ml/mouse, served as control group), estradiol-17β (100 ng/mouse), progesterone (2 mg/mouse), or a combination of the same doses of progesterone and estradiol-17β (E2+P4), respectively. At the same time, some mice were given with ICI 182780 (100 µg/mouse, Sigma) or RU486 (400 µg/mouse, Sigma) 1 h before injection of estradiol-17β and progesterone, respectively. Uteri from these mice were collected at 0, 3, 6, 12 and 24 hours after each treatment, and frozen into liquid nitrogen for further analysis.

### Isolation of uterine stromal cells and in vitro decidualization

Uterine stromal cells were isolated as previously described [23]. Briefly, uterine horns from mice on day 4 of pregnancy were dissected longitudinally and cut into small pieces (2–3 mm). Uterine samples were first digested in PBS containing 0.25% trypsin for 1 h at 4°C, followed by 1 h at room temperature. The digested uteri were shaken gently to remove sheets of luminal epithelial cells. The remaining tissues were washed twice in PBS and then placed in PBS containing 0.5% collagenase Type II for 30 min at 37°C. The digested uteri were vigorously shaken until the supernatant became turbid with dispersed stromal cells. The supernatant was passed through 75 and 38 mm strainers and centrifuged to obtain the stromal cells. Cell pellets were washed twice with the medium. Cells were then plated at 2×10^5^ cells per 6-well culture plates. After culture for 1 h, the medium was changed to remove unattached cells. Primary uterine stromal cells were induced for in vitro decidualization with phenol-red-free culture medium (DMEM/Ham's F-12, 1∶1) containing 10% charcoal-stripped FBS, 10 nM E2 and 1 µM P4, and collected at 24 h, 48 h and 72 h for further analysis.

### RNA isolation and quantitative PCR

Total RNA was extracted from uterine tissues or stromal cells with the TRIzol solution (Invitrogen, CA) according to the manufacturer's protocol. RNA (2 µg) samples were reverse-transcribed into single-stranded cDNA in a 25 µl reaction mixture (Promega, China). Real-time PCR was then performed in a 20 µl reaction volume containing 10 µl of 2× Brilliant SYBR Green Mix (TaKaRa, China), 2 µl of template cDNA, 0.5 µM primers, and 300 nM reference dye using the ABI thermal cycler 7500. The thermal cycling conditions were 95°C for 30 sec, followed by 40 cycles at 94°C for 5 sec, 60°C for 34 sec. Melting curve analysis and agarose gel electrophoresis were conducted following the quantitative PCR assays to monitor PCR product purity. The results were analyzed using ABI Prism 7500 software (Applied Biosystems, USA). 18 S was employed for normalization. The following primers were used: Kiss1: sense, 5′-CGAAGGAGTTCCAGTTGTAGG-3′,antisense,5′-AAGGAATCGCGGTATGCA-3′;GPR54:sense,5′-CCGTCCAACGCTTCAGGAT-3′,antisense,5′-GTGTAGCGAAAAACAGGGGAA-3′; decidual prolactin-related protein (dPRP): sense, 5′-TTATGGGTGCATGGATCACTC C-3′, antisense, 5′-CCCACGTAAGGTCATCATGGA T-3;18S: sense, 5′-AATCAG GGTTCGATTCCG GA—3′, antisense, 5′-CCA AGA TCCAACTACGAGCT-3′.

### Immunohistochemistry

Tissues were fixed in Bouin's solution, dehydrated, and embedded in paraffin. After tissue sections were deparaffinized and rehydrated in a graded series of ethanol solutions, endogenous peroxidase activity was blocked by incubating the sections in 3% hydrogen peroxide for 10 min. Nonspecific binding was blocked in 5% BSA for 60 min. Then, the sections were incubated in rabbit anti-kisspeptin (1∶150, MILLIPORE, USA) or rabbit anti-GPR54 (1∶300, Abcam, USA) overnight at 4°C. After washing in PBS, the sections were incubated with a secondary antibody for 45 min at 37°C. The primary antibody was detected with fresh diaminobenzidine solution, together with counter-staining with Harris' hematoxylin. In some sections, the primary antibodies were replaced with rabbit preimmune IgG as a negative control.

### Indirect immunofluorescence

Stromal cells were fixed in 4% paraformaldehyde for 20 min and permeated by using 0.1% Triton X-100 for 15 min at room temperature. Nonspecific binding was blocked in PBS with 5% BSA.Then, cells were incubated with mouse anti-human cytokeratin (Santa Cruz, USA) or mouse anti-human vimentin (Santa Cruz, USA) at 4°C overnight, followed by fluorescein isothiocyanate-labeled secondary antibody for 1 h at room temperature. Nuclei were stained with 5 g/ml propidiumiodide for 10 min. Finally, cells were viewed under a fluorescence microscope (Leica, Germany).

### siRNA transfections

Transfection assay was performed according to manufacturer's instructions. First, 2×10^5^ cells per well were cultured in 2 ml antibiotic-free normal growth medium for 24 h. Then, the growth medium was removed from wells, and 1 ml siRNA Transfection Medium (sc-36868, Santa Cruz, USA) were added to each well containing 6 µl Kiss1 siRNA duplex (sc-146490, Santa Cruz, USA) or 6 µl negative Control-siRNA (sc-36869, Santa Cruz, USA) and 8 µl siRNA Transfection Reagent (sc-29528, Santa Cruz, USA). After 7 h, aspirate transfection medium and replace with fresh normal growth medium, cells were cultured with normal medium and collected at 24 h, 48 h and 72 h for further analysis.

### Western blot analysis

Tissues and cells were lysed in the RIPA buffer supplemented with a phosphatase inhibitor cocktail (Applygen Technologies, China) and PMSF. A total of 20 µg protein extracts were subjected on the 12% SDS-polyacrylamide gel for electrophoresis, and then transferred onto the polyvinylidene difluoride membranes (Millipore, USA). The membranes were blocked with 5% nonfat milk for 1 hour at room temperature and incubated with the following primary antibodies at 4°C overnight: rabbit anti-Kisspeptin antibody(KISS1) (Abcam, USA), rabbit anti-progesterone receptor (PR) (Santa Cruz, USA), mouse anti-cyclin D3 (Cell Signaling, USA), and rabbit anti-β-actin(Santa Cruz, USA). The membranes were then incubated with horseradish peroxidase-conjugated secondary antibodies and visualized via enhanced chemiluminescence (Pierce, USA). The relative band intensity was acquired by using the Quantity One software. The data were corrected for background, normalized to β-actin expression.

### Statistical analysis

Each experiment was performed at least three times. Data were presented as the means ±SEM. The results were analyzed using one-way ANOVA, followed by an LSD post-hoc test, or using Student's t-test between two groups, and a value of P<0.05 was considered to be statistically significant. All statistical analyses were performed with SPSS13.0 program.

## Results

### Expression of Kiss1 and GPR54 in the uterus during mouse periimplantation

**Figure 1:**
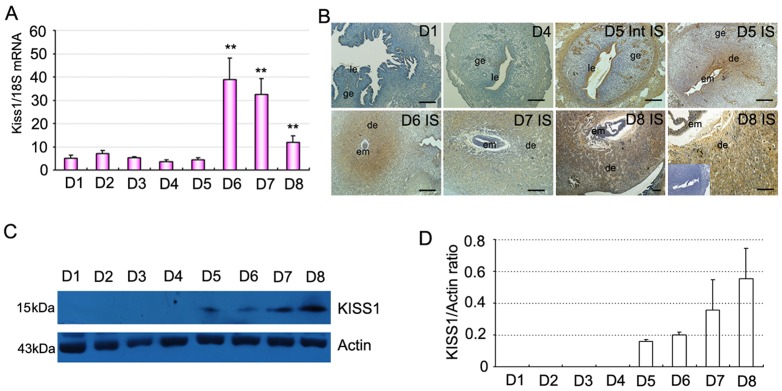
Expression of Kiss1 in mouse uterus during early pregnancy. (A) Levels of Kiss1 mRNA expression in the uterus during early pregnancy via quantitative PCR analysis. (B) Immunostaining of KISS1 protein in mouse uterus on days 1, 4, 5, 6, 7, and 8 of pregnancy. Inset: control (without primary antibody). (C) Representative immunoblotting results for KISS1 protein in mouse uterus on days 1–8 (D1–D8) of pregnancy. (D) Densitometric analyses of KISS1 protein in mouse uterus on days 1–8 of pregnancy. All experiments were repeated three times. Data are shown as means ± SEM. **p<0.01 compared with D1 of pregnancy. Scale bar, 50 µm. IS, implantation site; Int IS, inter-implantation site; em, embryo; ge, glandular epithelium; le, luminal epithelium; de, deciduas.

**Figure 2:**
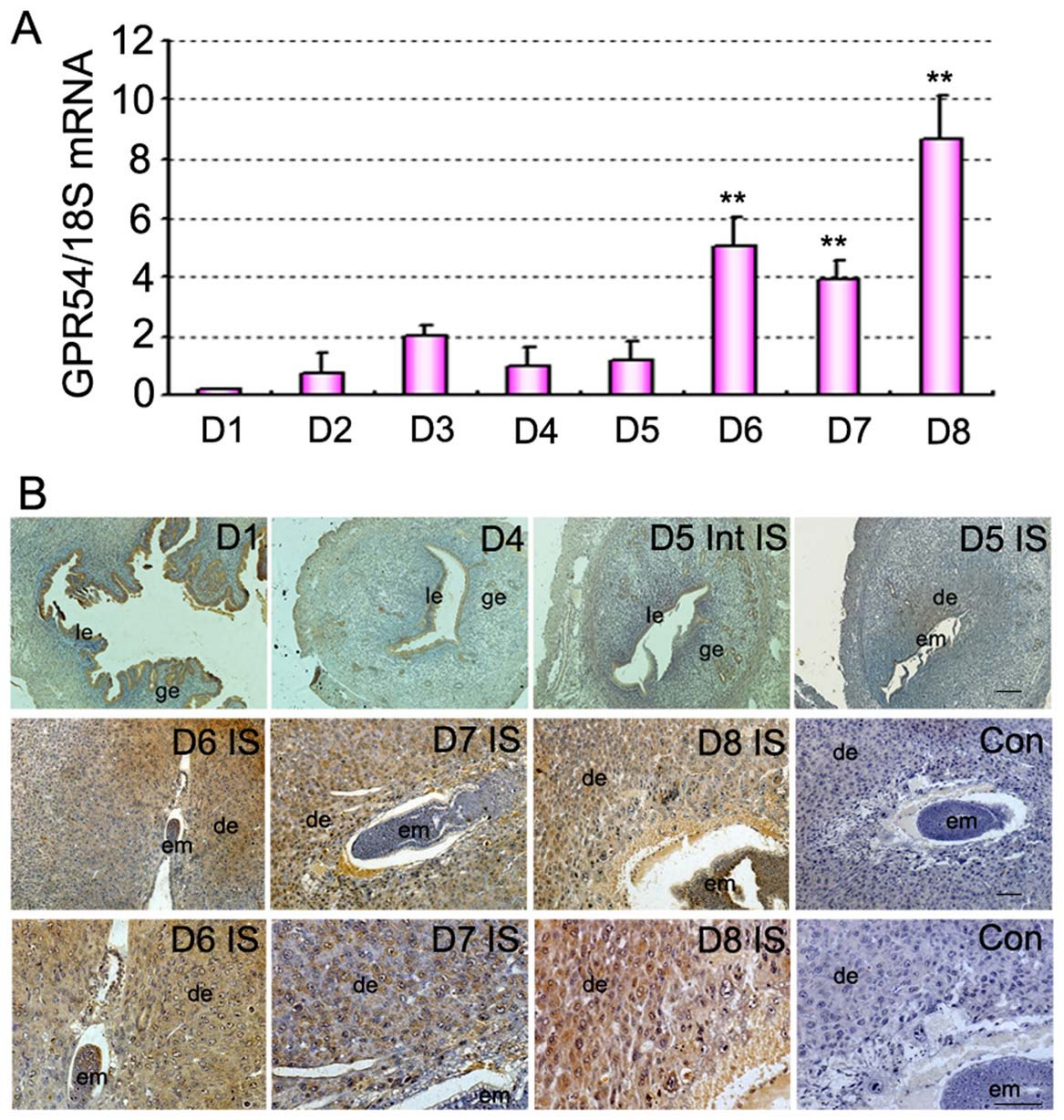
Expression of GPR54 in mouse uterus during early pregnancy. (A) Levels of GPR54 mRNA expression in the uterus during early pregnancy via quantitative PCR analysis. (B) Immunostaining of GPR54 protein in mouse uterus on days 1, 4, 5, 6, 7, and 8 of pregnancy. Upper and middle panel show original magnification ×100. Sections in middle panel were magnified in the bottom with higher magnification of ×200, respectively. All experiments were repeated three times. Data are shown as means ± SEM. **p<0.01 compared with D1 of pregnancy. Scale bar, 50 µm. Con, control: without primary antibody; IS, implantation site; Int IS, inter-implantation site; em, embryo; ge, glandular epithelium; le, luminal epithelium; de, deciduas.

Expressions of Kiss1 and GPR54 mRNA in mouse uterus during periimplantation period were first examined by quantitative PCR as showed in Figure 1A and 2A. From D1 to D5 of pregnancy, Kiss1 and GPR54 expression levels were generally low. In contrast with D1, the expression level of Kiss1 and GPR54 mRNA dramatically increased with the progression of uterine decidualization (days 6–8; Fig. 1A, 2A; P<0.01). Furthermore, Western blot analyses revealed a significant increase expression of KISS1 protein in the uterus with the initiation of implantation (day 5 vs. days 1–4) and decidualization (days 6–8; Fig. 1C, D). We then analyzed the spatiotemporal expression of KISS1 and GPR54 protein in mouse uterus on days 1–8 of pregnancy by immunohistochemistry (Fig. 1B, 2B). The results showed that from D1 to D4 of pregnancy, KISS1 and GPR54 protein were major located in the luminal and glandular epithelium with low level in accordance with quantitative RT-PCR results (Fig. 1A, 2A). While from D5 onward, the expression of GPR54 and especially KISS1 protein significantly increased and was restricted in the decidualizing stromal cells surrounding the implanted embryo (Fig. 1B, 2B). By contrast, in the inter-implantation sites on day 5 of pregnancy, KISS1 and GPR54 proteins were visualized in the epithelium and myometrium (Fig. 1B, 2B).

### Expression of Kiss1 and GPR54 are associated with uterine decidualization independent of the presence of embryo

**Figure 3:**
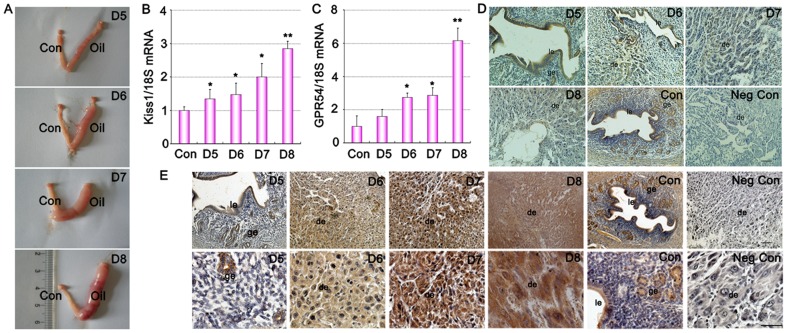
Expression of Kiss1 and GPR54 in mouse uterus during artificially induced decidualization in vivo. (A) Representative uteri of oil-induced decidualization on days 5–8 of pseudopregnant. Con, uninjected oil horn served as a control; Oil, inject oil horn. (B) Quantitative PCR analysis of Kiss1 mRNA in oil-induced decidualization uteri on days 5, 6, 7, and 8 of pseudopregnancy. (C) Quantitative PCR analysis of GPR54 mRNA in oil-induced decidualization uteri on days 5, 6, 7, and 8 of pseudopregnancy. (D) Immunostaining of KISS-1 protein in oil-induced decidualization uteri on days 5, 6, 7, and 8 of pseudopregnancy. (E) Immunostaining of GPR54 protein in oil-induced decidualization uteri on days 5, 6, 7, and 8 of pseudopregnancy. Upper panel show original magnification ×100. Sections in upper panel were magnified in the bottom with higher magnification of ×400, respectively. All experiments were repeated three times. Data are shown as means ± SEM. *p<0.05, **p<0.01 compared with control. Scale bar, 50 µm. Negative control, without primary antibody; Con, control; ge, glandular epithelium; le, luminal epithelium; de, deciduas.

To examine whether Kiss1 and GPR54 expression are dependent on the presence of living embryo or just induced by uterine decidualization reaction, we analyzed Kiss1 and GPR54 expression in a well utilized model of artificial uterine decidualization by oil induction (Fig. 3 A). As shown in figure 3, Kiss1 and GPR54 expression patterns are strikingly similar with that observed in normal implantation and decidualization (Fig. 1 and 2), becoming apparent in the artificially induced decidualizing stromal cells on days 6–8 of pseudopregnancy (Fig. 3 D–E). These results suggested that Kiss1 and GPR54 were involved in the stromal cell decidualization.

### Effects of steroid hormones on Kiss1 expression

**Figure 4:**
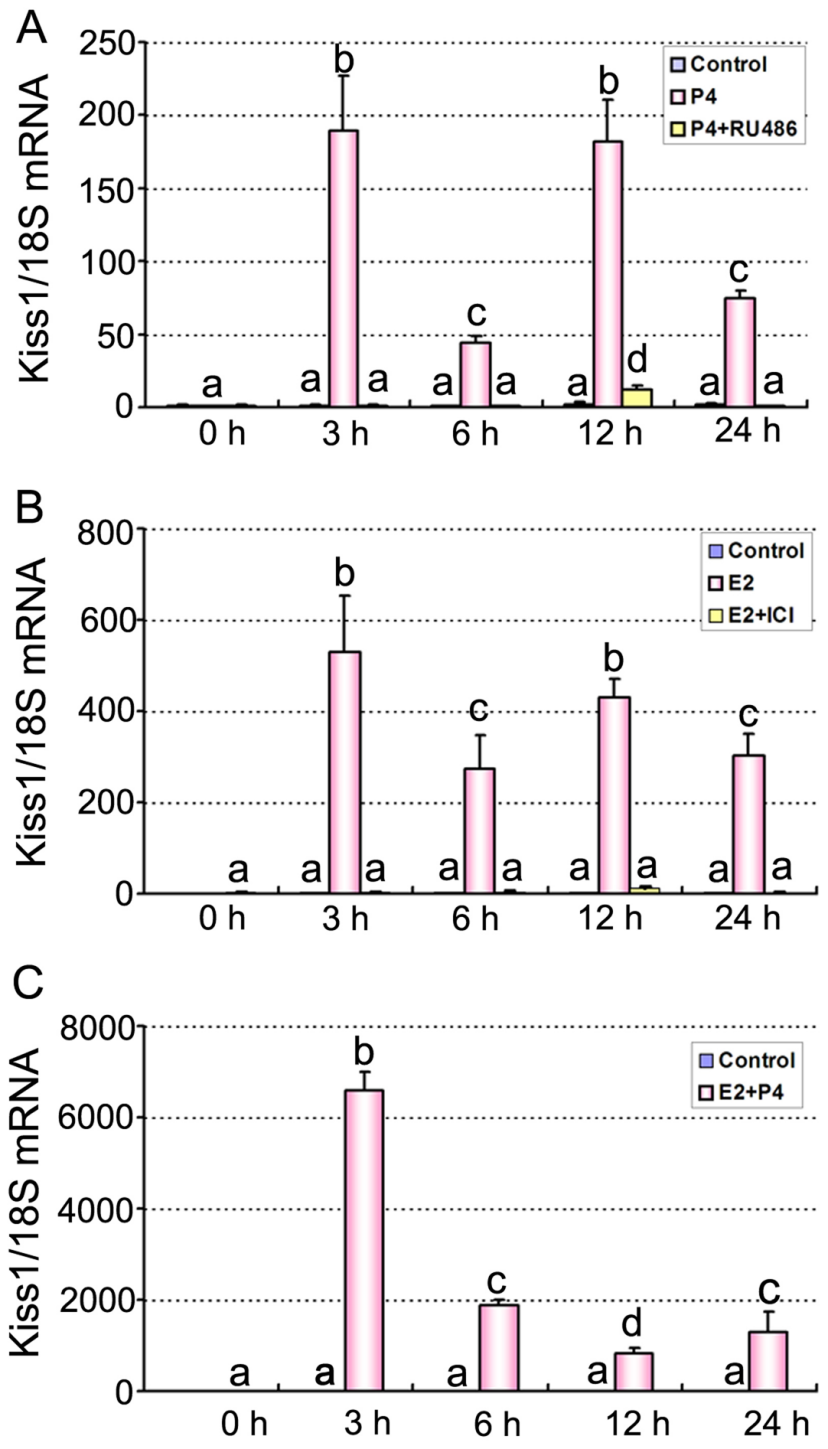
Quantitative PCR analysis of Kiss1 mRNA in mouse uteri after steroid hormone treatments. (A) Expression of Kiss1 mRNA in mouse uteri at 0, 3, 6, 12, and 24 h after P4 plus RU486 treatment. (B) Expression of Kiss1 mRNA in mouse uteri at 0, 3, 6, 12, and 24 h after E2 plus ICI 182780 treatment. (C) Expression of Kiss1 mRNA in mouse uteri at 0, 3, 6, 12, and 24 h after P4 plus E2 treatment. Mice injected with oil (vehicle) served as controls. Data are shown as means ± SEM (n = 3). Groups with different superscript letters are significantly different (ANOVA followed by LSD multiple range test).

There was low or no detectable Kiss1 mRNA signals in the ovariectomized uterus (Fig. 4). After the ovariectomized mice were treated with P4 (Fig. 4 A), E2 (Fig. 4 B) or a combination of P4 and E2 (Fig. 4 C), Kiss1 mRNA level significantly increased compared with sesame oil treatment (control group), with a peak at 3 h post-injection (Fig. 4). The induction was significantly blocked by co-treatment of PR antagonist RU486 (Fig. 4 A) and E2 antagonist ICI 182780 (Fig. 4 B).

### Increase of Kiss1 and GPR54 expression during decidualization of stromal cells in vitro

**Figure 5:**
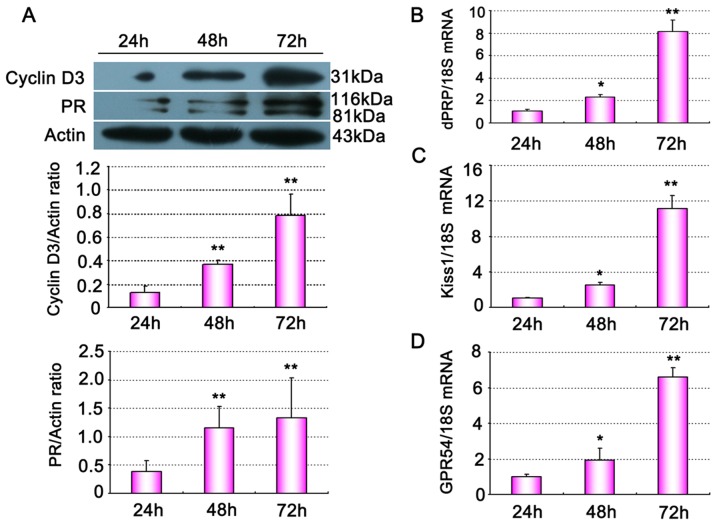
Gene expression of mouse uterine stromal cells undergoing decidualization in vitro. (A) Western blot analysis of cyclinD3 and PR proteins expression in the stromal cells cultured up to 72 h. (B–D) Quantitative PCR analysis of dPRP (B), Kiss1(C) and GPR54 (D) mRNA expression in stromal cells cultured up to 72 h. All experiments were repeated three times. Data are shown as means ± SEM. *p<0.05, **p<0.01 compared with 24 h group.

To explore the role of kisspeptin in vitro decidualization of mouse uterine stromal cells, stromal cells were firstly isolated from sensitized uteri on day 4 of pregnancy. The purity of isolated uterine stromal cells was monitored by positive staining of vimentin and negative staining cytokeratin (Figure S1A). 24–72 h after P4 and E2 combinative treatment, stromal cells exhibited morphological characteristics of polyploidy decidual cell, an indicator of terminal differentiation of stromal cells (Figure S1B). Western blot and quantitative PCR analysis showed that markers of decidualization including cyclin D3, PR (Fig. 5A), and dPRP (Fig. 5B) significantly increased at 48 and 72 h compared with 24 h in vitro culture. Quantitative PCR analysis found that Kiss1 (Fig. 5C) and GPR54 mRNA (Fig. 5D) levels were also gradually up-regulated in these cells during decidualization in vitro.

### Knockdown of Kiss1 attenuates decidualization of stromal cells in vitro

**Figure 6:**
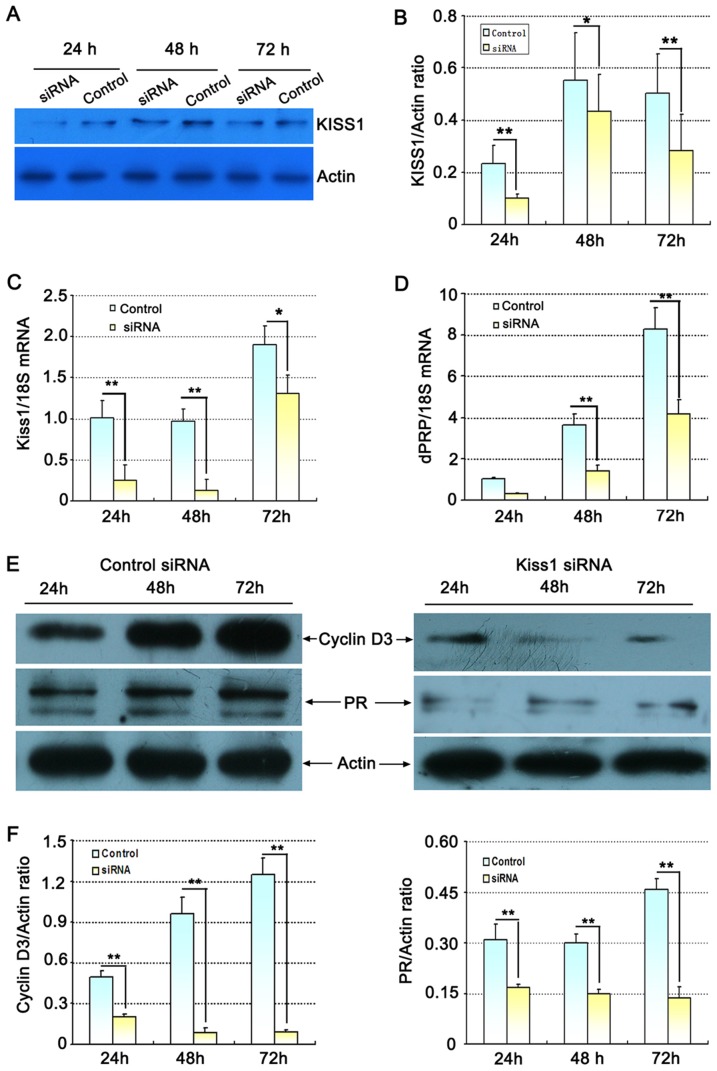
Effect of Kiss1 siRNA on the decidualization of uterine stromal cells in vitro. (A) Representative immunoblotting results for KISS1 protein following Kiss1 siRNA transfections. (B) Densitometric values from western blot analyses of KISS1 protein. (C) Expression of Kiss1 mRNA following Kiss1 siRNA transfections by quantitative PCR analysis. (D) Effect of Kiss1 siRNA on dPRP mRNA expression by quantitative PCR analysis. (E) Effect of Kiss1 siRNA on cyclinD3 and PR proteins expression via western blot analysis. (F) Densitometric values from western blot analyses of cyclinD3 and PR protein. All experiments were repeated three times. Data are shown as means ± SEM. *p<0.05, **p<0.01 compared with control siRNA.

In order to assess the function of Kiss1 in the decidualization of stromal cells, we established stromal cells that express either control non-target (control) or Kiss1-targeting small interfering RNA (siRNA). As shown in Fig. 6A–C, Kiss1 protein (Fig. 6 A, B) and mRNA (Fig. 6 C) in stromal cells was significantly decreased after transfection with Kiss1 siRNA compared with control group at 24, 48 and 72 h in vitro culture (P<0.01 or <0.05). This was accompanied by significant decrease in dPRP (mRNA; Fig. 6D), cyclin D3 (protein; Fig. 6E, F) and PR (protein; Fig. 6E, F) in the Kiss1 siRNA group in comparison with control-siRNA. These results provided an evidence that kisspeptin was essential for normal stromal cell decidualization.

## Disscussion

The uterine decidualization is crucial to normal pregnancy establishment in mice and humans [4], [24]. However, the molecular mechanism underlying this process remained largely unknown. In this study, the decidualization-specific expression pattern of Kiss1 and GPR54 during normal pregnancy indicated that Kiss1/GPR54 system might play a role in the decidualization of stromal cells. Moreover, Kiss1 expression is independent of the presence of living embryo, but up-regulated with the progression of stromal cell decidualization both in vivo and in vitro models. In addition, in the ovariectomized mouse uterus, the expression of Kiss1/GPR54 mRNA was also upregulated after progesterone or/and estradiol treatment. The attenuated expression of Kiss1 after siRNA knockdown significantly blocks the progression of decidualization.

During human pregnancy, circulating kisspeptin levels increased by 940-fold in the first trimester and further increased to some ∼7,000-fold higher in the third trimester compared with non-pregnant control [16]. Bilban M *et al* [17] demonstrated that circulating kisspeptin was major derived from the placenta and could inhibit the migration and invasion of human trophoblasts. Kiss1 and GPR54 gene expression are highly expressed in the trophoblast compartment of placenta [16], [17], [21]. Maternal decidua was devoid of Kiss1 mRNA staining [17]. In rodent, whether circulating kisspeptin levels remarkably increase in pregnancy remains unknown [6]. Terao Y et al demonstrated that Kiss1 and GPR54 mRNA were expressed in the trophoblast giant cells of the rodent placenta at embryonic day 12.5 by using in situ hybridization [21]. Our results revealed that Kiss1/GPR54 mRNA levels significantly increase in the mouse uterus during decidualization, and this does not depend on the presence of a conceptus. Kiss1/GPR54 proteins were spatiotemporally expressed in decidualizing stromal cells in intact pregnant females, as well as in pseudopregnant mice undergoing artificially induced decidualization. The expression discrepancy of Kiss1 and GPR54 at the maternal-fetal interface may be partly caused by species-specific differences and assay methods. In human, the decidualization of stroma cells begins before the embryo implantation during the late secretory phase, which is supportive to the implantation of blastocyst [25]; while in mouse, the decidualization process only happens after the initiation of implantation. Our results showed that Kiss1 mRNA was dynamically up-regulated only after the initiation of embryo implantation since day5 (when decidualization first began), suggesting its expression is tightly related to the temporal existence to mouse decidualization, which might be unique to the murine species.

In rodents, estrogen and progesterone are specifically required for the decidualization process of stromal cells, which comprises morphogenetic, biochemical and vascular changes driven by estrogen and progesterone receptors [4]. We have shown that the expression of Kiss1 in uteri was upregulated by P4 or/and E2 treatment in ovariectomized mice. This induction was significantly blocked by co-treatment of PR antagonist RU486, suggesting it is primarily mediated by nuclear progesterone receptor. However, co-treatment of E2 and tamoxifen further stimulated the expression of Kiss1 gene (data not shown). It is possible because tamoxifen is selective estrogen receptor modulators, which has agonistic and antagonistic dual role, and this effect depends on the different germline type of organization in the different target organs or cells [26], [27]. Such as tamoxifen has antagonistic role of estrogen in breast tissue, whereas it produces agonistic effects in the uterus and bone tissue [26], [27].

Currently, little is known about the function of Kiss1/GPR54 expression in the mouse or human uterus during uterine sensitization and decidualization. Kiss1 knockout mice were observed histological abnormalities in the uterus, showing thread-like uteri [28]. Female GPR54 null mice also displayed hypoplastic uterine horns [14], [15]. These data suggested that Kiss1/GPR54 system was involved in the growth and proliferation of uterine endometrium. Due to its global effect by affecting the pituitary-gonadal axis, the local effect of Kiss1/GPR54 system on uterine tissue remains unknown. In this study, culture models of uterine stromal cells have used to assess the function of Kiss1/GPR54 expression during the decidualization of mouse. We found that Kiss1/GPR54 expression significantly increases during the decidualization of stromal cells. Suppressing Kiss1 expression by siRNA may cause a decrease in the decidualization of stromal cells as indicated by the reduction of the expression of markers of decidualization, accompanied by significant decrease in dPRP, cyclin D3 and PR expressions. These results provided an evidence that kisspeptin was involved in regulating uterine decidualization.

The decidualization of uterine stromal cell is characterized by stromal cell proliferation and their differentiation into decidual cells with polyploidy [29]. By using MTS assay, we have shown that Kiss1 has no significant effect on the proliferation of stromal cell after Kiss1-siRNA treatment (data not shown). It suggested that Kiss1/GPR54 system might therefore function in decidualization through the regulation of cell differentiation process. Kisspeptin could regulate stromal cell differentiation by cyclin D3 protein which plays a specific role in stromal cell polyploidy [29], but the detailed process of regulation remains unknown. On the other hand, dPRP is not only a useful indicator of decidualization[23], but also an excellent marker for the differentiated antimesometrial cells[30], and it stimulates vascular endothelial cell proliferation[31]. Our results showed that Kiss1 could stimulate the expression of dPRP during the process of decidualization. Therefore, Kiss1 might function in decidualization through the regulation of cell differentiation and angiogenesis of endometrium. Although this study suggests that Kiss1/GPR54 system plays a key role in decidualization of mouse, the identity of downstream target genes of Kiss1 need to be elucidated further.

In conclusion, this study demonstrated that Kiss1/GPR54 system was spatially and temporally expressed in mouse uterus during early pregnancy and decidualization, which was regulated by steroid hormones and decidualization. Furthermore, our results suggested that kisspeptin may play a key role in the decidualization of mouse stromal cells.

## Supporting Information

Figure S1 **The vimentin and cytokeratin Immunostaining and morphology changes of isolated uterine stromal cells.** (A) Immunofluorescence analysis of vimentin and cytokeratin in primary stromal cells. Green signal represents vimentin staining with FITC-conjugated secondary antibody and red signal indicates nuclear staining with PI. Control, without primary antibody. (B) Morphology and decidual polyploidization of stromal cells cultured up to 72 h. hematoxylin analysis of decidual polyploidization after cultured 24 h, 48 h, and 72 h in vitro. Scale bar, 50 µm.
(TIF)
