# Supplementary figures and images for: Expression and Function of Kisspeptin during Mouse Decidualization
Source: PLoS One. 2014 May 15;9(5):e97647. doi: 10.1371/journal.pone.0097647 (PMC4022638; doi:10.1371/journal.pone.0097647)

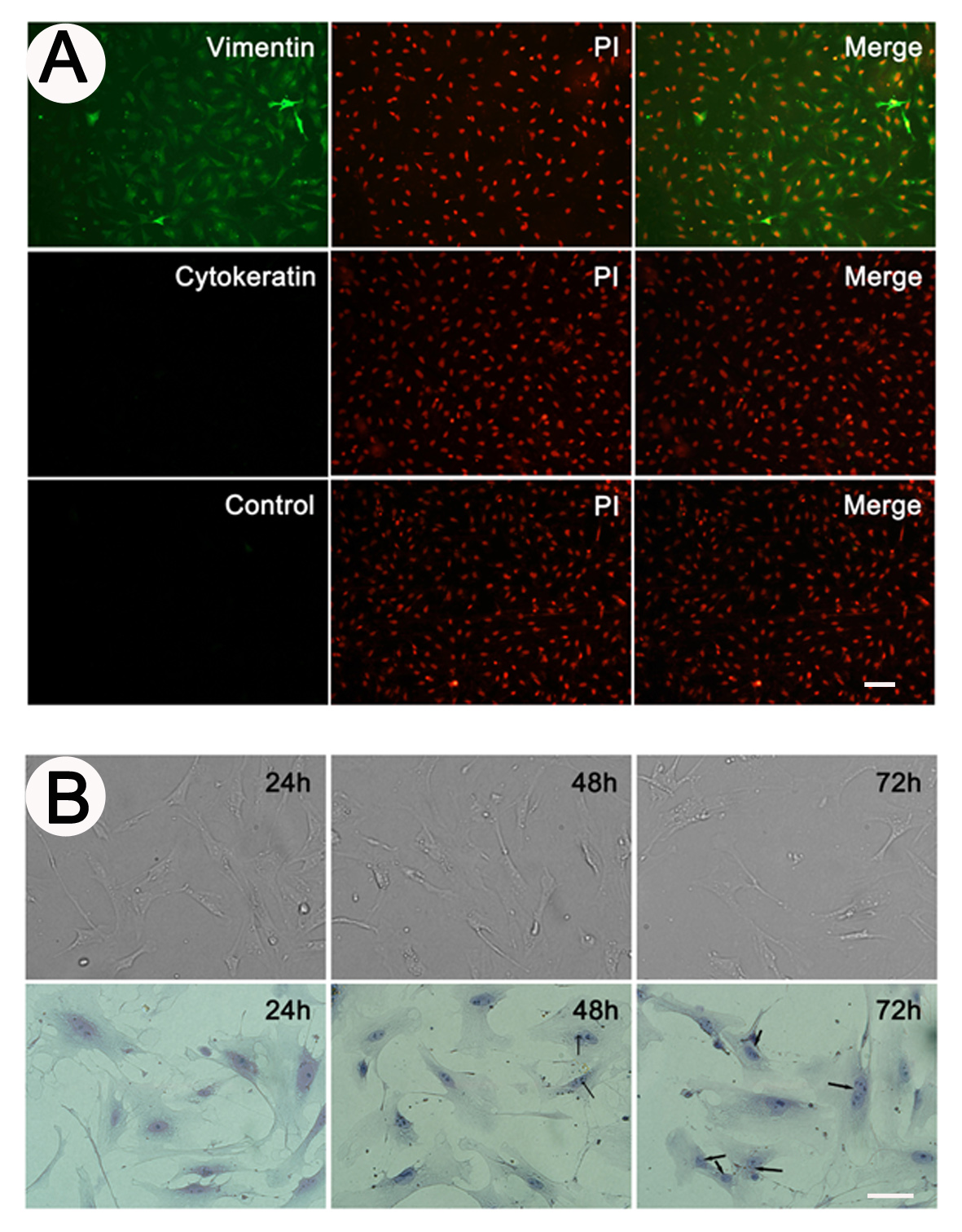

Supplement: Figure S1 — The vimentin and cytokeratin Immunostaining and morphology changes of isolated uterine stromal cells. (A) Immunofluorescence analysis of vimentin and cytokeratin in primary stromal cells. Green signal represents vimentin staining with FITC-conjugated secondary antibody and red signal indicates nuclear staining with PI. Control, without primary antibody. (B) Morphology and decidual polyploidization of stromal cells cultured up to 72 h. hematoxylin analysis of decidual polyploidization after cultured 24 h, 48 h, and 72 h in vitro. Scale bar, 50 µm. (TIF) [file pone.0097647.s001.tif]
